# Optimizing Color-Difference Formulas for 3D-Printed Objects

**DOI:** 10.3390/s22228869

**Published:** 2022-11-16

**Authors:** Min Huang, Xinyuan Gao, Jie Pan, Xiu Li, Caroline Hemingray, Kaida Xiao, Manuel Melgosa

**Affiliations:** 1 Printing and Packaging Engineering, Beijing Institute of Graphic Communication, Beijing 102600, China; 2 School of Design, University of Leeds, Leeds LS2 9JT, UK; 3 Optics Department, Faculty of Sciences, University of Granada, 18071 Granada, Spain

**Keywords:** color difference, 3D color printing, CIEDE2000, CIECAM02, CIECAM16

## Abstract

Based on previous visual assessments of 440 color pairs of 3D-printed samples, we tested the performance of eight color-difference formulas (CIELAB, CIEDE2000, CAM02-LCD, CAM02-SCD, CAM02-UCS, CAM16-LCD, CAM16-SCD, and CAM16-UCS) using the standardized residual sum of squares (*STRESS*) index. For the whole set of 440 color pairs, the introduction of *k_L_* (lightness parametric factor), *b* (exponent in total color difference), and *k_L_* + *b* produced an average *STRESS* decrease of 2.6%, 26.9%, and 29.6%, respectively. In most cases, the CIELAB formula was significantly worse statistically than the remaining seven formulas, for which no statistically significant differences were found. Therefore, based on visual results using 3D-object colors with the specific shape, size, gloss, and magnitude of color differences considered here, we concluded that the CIEDE2000, CAM02-, and CAM16-based formulas were equivalent and thus cannot recommend only one of them. Disregarding CIELAB, the average *STRESS* decreases in the *k_L_* + *b*-optimized formulas from changes in each one of the four analyzed parametric factors were not statistically significant and had the following values: 6.2 units changing from color pairs with less to more than 5.0 CIELAB units; 2.9 units changing the shape of the samples (lowest *STRESS* values for cylinders); 0.7 units changing from nearly-matte to high-gloss samples; and 0.5 units changing from 4 cm to 2 cm samples.

## 1. Introduction

In recent years, due to the development of three-dimensional (3D) color printing technology, much attention has been given to the color reproduction quality of 3D-printed objects [1,2]. Consequently, color measurement and color-difference evaluation of 3D sample pairs is becoming increasingly important in color reproduction and color control processes. In fact, a variety of color-difference formulas are currently used in both research and industrial applications to measure perceived color differences between sample pairs, while the International Commission on Illumination (CIE) has requested new reliable visual datasets to develop more accurate color-difference formulas [3]. Among these formulas, we can mention the well-known CIELAB formula [4], the CIEDE2000 formula currently recommended by the ISO and CIE [5], and some formulas based on the two latest color-appearance models proposed by the CIE: CAM02-(LCD/SCD/UCS) [6], which is based on CIECAM02 [7]; and CAM16-(LCD/SCD/UCS) [8], which is based on CIECAM16 [9]. It is worth noting, however, that all color-difference formulas currently employed were developed or tested using only datasets with flat (2D) color samples such as self-illuminated visual displays, papers, textiles, plastics, etc. [10,11]. Compared with 2D samples, the visual color perception of 3D samples is more complicated and may be affected by factors such as shape [12], the geometrical structure of the lighting [13], translucency [14], gloss [15,16], etc. Visual differences and applicability of current color-difference formulas for 3D sample pairs must be more carefully studied, as is currently being done by CIE Technical Committee 8–17, “Methods for Evaluating Color Difference between 3D Color Objects” [17]. Hopefully, a successful prediction of visual color differences for 3D objects will be useful for industry and in different applications [18,19,20,21].

Jiang et al. [22] conducted a study to evaluate color differences for 3D and 2D objects using an Mcor Iris paper-based 3D color printer. Seventy-five pairs of both spherical 3D samples and flat 2D samples were prepared, and their color differences were assessed by ten observers using the gray scale method. All 3D spheres had the same gloss and size, with the average color difference of sample pairs being relatively high (10.3 CIELAB units). The performances of 10 color-difference formulas (CIELAB, CMC, CIEDE2000, CAM02, DIN99d, etc.) were then analyzed; the results indicated that the CAM02-LCD formula provided the best performance.

He et al. [23] invited 15 observers to carry out color-difference evaluations for 82 pairs of 3D spherical samples using the gray scale method. The average color differences of their Experiments I and II were 5.5 and 4.6 CIELAB units, respectively. The CIELAB and CIEDE2000 formulas were optimized using different methods; it was found that for both formulas, the predictions of the visual results were improved by using optimized lightness parametric factors but not by using optimized power functions.

Huang et al. [24] performed a large set of visual experiments using 440 pairs of 3D-printed samples with three different shapes (spheres, cones, and cylinders), two different sizes (4 cm and 2 cm), and two materials (matte and glossy). The color differences in these experiments were in a large range (0.5–16.9 CIELAB units). Forty-five color normal observers participated in these experiments using the gray scale method. The main goal of the current paper was to use the results of these recent visual experiments [24] to evaluate and optimize the performances of eight color-difference formulas: CIELAB [4], CIEDE2000 [5], CAM02-(LCD/SCD/UCS) [6] and CAM16-(LCD/SCD/UCS) [8]. The CAM16-(LCD/SCD/UCS) formulas are analogous to the CAM02-(LCD/SCD/UCS) formulas, using as a starting point the CIECAM16 color appearance model [8] in place of the CIECAM02 model [7]. Here, the letters LCD and SCD mean “large” and “small” color differences, respectively, while the letters UCS mean “uniform color scale” and refer to a general-purpose color-difference formula that was intermediate between the SCD and LCD formulas.

In the current paper, we used the standardized residual sum of squares (*STRESS*) index recommended by the CIE [25] to measure the agreement between predictions made by these models and the average visual results from observers with normal color vision reported in [24]. Regarding optimizations of the above-mentioned color-difference formulas, following the previous literature [22,23] we used the lightness parametric factor (*k_L_*) already proposed in the CIEDE2000 formula [7], and power functions [26,27]. The use of parametric factors in color-difference formulas has been recommended to account for changes with respect to “reference conditions”, which are defined as the set of illuminating and viewing conditions recommended in the use of a color-difference formula. In our case, the use of 3D objects, with their related consequences, was the main change with respect to the “reference conditions” recommended for CIEDE2000 [5]. On the other hand, the use of power functions in color-difference formulas has been mainly proposed to account for non-linearities of perceived color differences in a wide range of magnitude [28]. The influences of four different parameters (color difference magnitude, gloss, shape, and size) on the performances of the eight mentioned color-difference formulas are also analyzed in this paper for the case of the specific 3D objects considered in [24], complementing the results found for 2D objects [29].

## 2. Methodology

### 2.1. Characteristics of 3D Color Pairs 

In the current study, we tested and optimized eight specific color-difference formulas using the visual color-difference data from Huang et al. [24] for 3D objects of different shapes, sizes, and degrees of gloss. These 3D objects were printed using Sailner J400 and J501 3D color printers (Sailner 3D Technology Col., Ltd., Zhuhai, China), had colors located around five CIE-recommended centers, and were very homogeneous (i.e., they had no visual perceptible texture, surface roughness, graininess, or sparkle), as shown in Figure 2 in [24]. Table 1 summarizes the main characteristics of the four experiments in [24], which were named Experiments I–IV; these are separated by horizontal lines. According to the shape, size, and material (gloss/matte) of the 3D samples employed in these visual experiments, there were eight phases, namely Sp-4-m, Sp-4-g, Sp-2-m, Sp-2-g, Co-4-m, Co-4-g, Cy-4-m, and Cy-4-g, as shown in column 2 of Table 1. In these designations, the two initial letters indicate the shape of the samples (Sp, Co, and Cy for spheres, cones, and cylinders, respectively); the number 4 or 2 indicates the size (the diameter of the spheres or the bottom diameter and height of the cones and cylinders) of the samples in centimeters; and the letters m or g indicate the surface of the material, which will be briefly designated here as matte or glossy, respectively. For example, Sp-4-m indicates the color-difference experiments that used pairs of matte spheres with a 4 cm diameter. The last row in Table 1 (phase 9) provides information on the entire dataset obtained by combining the color pairs of the eight phases provided in [24]. The average values of the matte and glossy samples used in the experiments described in [24] were 3.6 GU and 96.6 GU, respectively, based on measurements at 60° made using a TC-108DPA gloss meter (Tokyo Denshoku Co. Ltd., Tokyo, Japan) in such a way that they could be considered as nearly matte and high-gloss color samples. All of the visual experiments described in [24] were carried out in a dark room using the gray scale method and 26–45 observers with normal color vision. The gray scales employed in these visual experiments had 14 color pairs that were viewed by the observers together with the 3D color pairs placed at a distance of approximately 40 cm and using viewing cabinets with specific light sources, as described in Section 2 of [24]. The visual angles subtended by color pairs at each observer’s position was greater than 4°, so the CIE 1964 standard colorimetric observer was used in the computations of the color coordinates. Instrumental color measurements of all samples were performed using an X-Rite Ci64 spectrophotometer in SCI mode. The gray scale values reported by the observers were transformed to visual color differences using specific equations derived from the CIELAB color differences of the color pairs in gray scale, as described in Section 2.E of [24]. To compute the intra-observer variability, several observers replicated their visual assessments, as can be concluded based on the numbers shown in the last column of Table 1.

**Figure 1:**
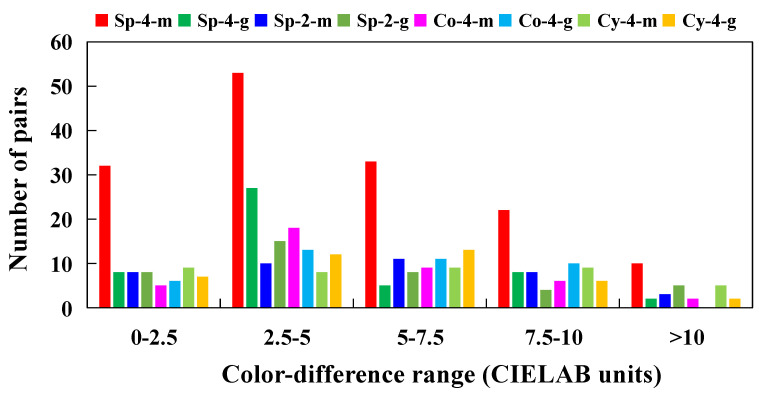
Numbers of pairs with different color-difference magnitudes for each of the 8 phases (see main text).

The ranges and average values of the CIELAB color differences for the 440 pairs of samples employed in [24] are shown in columns 6 and 7 of Table 1, respectively. As can be seen, these pairs of samples had suprathreshold color differences in a considerably large range up to 16.9 CIELAB units. The number of color pairs (and percentages) in the ranges of 0.0–2.5, 2.5–5.0, 5.0–7.5, 7.5–10.0, and beyond 10.0 CIELAB units were 83 (19%), 156 (35%), 99 (23%), 73 (17%), and 29 (7%), respectively. More specifically, Figure 1 shows the number of color pairs in these ranges for each of the eight phases (see Table 1). In Table 1 we can note that for any phase there were at least 40 color pairs and 26 observers. By putting together the results of the eight phases in [24], we obtained a combined dataset of 440 color pairs with a total of 20,170 visual assessments (the last row in Table 1), which may be particularly useful for the testing and optimization of color-difference formulas for 3D objects, as intended in the current paper.

### 2.2. Tested Color-Difference Formulas

We tested the performances of eight color-difference formulas (CIELAB [4], CIEDE2000 [5], CAM02-(LCD/SCD/UCS) [6], and CAM16-(LCD/SCD/UCS) [8]) with respect to the visual data reported in [24] while considering both the original and optimized formulas (see Section 2.3). It should be noted that these eight color-difference formulas included CIEDE2000—the current ISO/CIE recommended formula—and pertain to the following three main groups: formulas based on CIELAB [4], CIECAM02 [7], and CIECAM16 [9]. In the current study, the values for the adapting luminance, background luminance, and surround in the color-difference formulas CAM02-(LCD/SCD/UCS) [6] and CAM16-(LCD/SCD/UCS) [8] were 100, 20, and average, respectively, according to viewing conditions in the visual experiments in [24]. 

The standardized residual sum of squares (*STRESS*) index that is currently recommended by CIE [25] was used to compare the performances of the different color-difference formulas tested. The usefulness of the *STRESS* index was recently revised [30]; in our case, it was reasonable to maintain it because the minimum number of color pairs in any phase was considerably high (≥40). The values of the *STRESS* index are always in the range of 0–100. The smaller the *STRESS* value, the better the agreement is between visual results and predictions made according to a given color-difference formula. Using F-tests from ratios of squared values of *STRESS* indices [25], it is possible to know whether two color-difference formulas are significantly different statistically for a given set of visual data or not. Note that the results from F-tests are particularly interesting because the proposal of a new color-difference formula is only acceptable when it is significantly better statistically than previous ones for a wide set of reliable visual data. In the current study, we used F-tests while considering two-tailed *F*-distributions with a 95% confidence level. To evaluate the merit of a given color-difference formula, here we also arbitrarily assigned a score of 10 points to the formula when it was significantly better statistically than another (7.5 points if it was only better, 5 points if it was identical, 2.5 points if it was worse, and 0 points if it was significantly worse than another). Considering a visual dataset (e.g., the one in phase 9 of Table 1), the eight color-difference formulas tested were ranked using the average score of the given formula in comparison with the seven remaining ones.

The *STRESS* index can also be used to measure intra- and inter-observer variability [31]. The mean intra- and inter-observer variability in the experiments in [24] was in the range of 16.7–21.9 and 30.3–31.2 *STRESS* units, respectively. In general, a color-difference formula is acceptable when it provides *STRESS* values lower than inter-observer variability.

### 2.3. Optimizations of Color-Difference Formulas

We analyzed the improvements produced in each of the eight mentioned color-difference formulas by introducing two different modifications: (1) optimization of the value of the lightness parametric factor (see Section 2.3.1); and (2) optimization of the value of an exponent (power function) added to the original color-difference formula (see Section 2.3.2). Section 3 shows the results found when these two modifications were applied separately and simultaneously.

#### 2.3.1. Optimization of the Lightness Parametric Factor (*k_L_*)

Assuming the CIE 1964 standard colorimetric observer, Equation (1) shows the CIELAB color-difference formula, while the general format of most CIELAB-based color-difference formulas (e.g., CIEDE2000) is shown in Equation (2). In these equations, $\Delta L_{ab,10}^{*}$, $\Delta C_{ab,10}^{*}$, and $\Delta H_{ab,10}^{*}$ are the lightness, chroma, and hue differences [4], respectively; *k_L_*, *k_C_*, and *k_H_* are three constants called the lightness, chroma, and hue parametric factors, respectively; and ΔR is a chroma–hue interaction term that was called the “rotation term” in CIEDE2000 [5].

(1) $$\Delta E_{ab,10}^{*}=\sqrt{\Delta L_{ab,10}^{*}{}^{2}+\Delta C_{ab,10}^{*}{}^{2}+\Delta H_{ab,10}^{*}{}^{2}}$$

(2) $$\Delta E_{1}=\sqrt{{\left(\frac{\Delta L_{ab,10}^{*}}{k_{L}}\right)}^{2}+{\left(\frac{\Delta C_{ab,10}^{*}\;}{k_{C}}\right)}^{2}+{\left(\frac{\Delta H_{ab,10}^{*}\;}{k_{H}}\right)}^{2}+\Delta R}$$

CIE recommends *k_L_* = *k_C_* = *k_H_* = 1 for CIEDE2000 under the so-called “reference conditions”, which implicitly assumed the use of 2D object samples as well as *k_L_* = 2 for textile samples [5]. The reasons for this last recommendation are unknown. In some previous papers, researchers optimized the values of the parametric factors to improve the predictions made using Equation (2) while paying particular attention to the optimization of the lightness parametric factor *k_L_* [23,29,32,33,34,35,36], as was done in the current paper.

Equation (3) shows the common format of the CAM02-(LCD/SCD/UCS) and CAM16-(LCD/SCD/UCS) color-difference formulas, where $J^{\prime }$ (lightness), $a^{\prime }$, and $b^{\prime }$ are the three coordinates of the color spaces based on CIECAM02 and CIECAM16 [6,8], respectively, and $k_{L}$ is the lightness parametric factor to be optimized:

(3) $$\Delta E_{2}=\sqrt{{\left(\frac{\Delta J^{\prime }}{k_{L}}\right)}^{2}+\Delta {a^{\prime }}^{2}+\Delta {b^{\prime }}^{2}}$$

More specifically, the proposed values of $k_{L}$ for the CAM02-LCD, CAM02-SCD, and CAM02-UCS formulas were 0.77, 1.24, and 1.0, respectively [6].

#### 2.3.2. Optimization of Exponent *b* (Power Function)

The use of power functions was previously proposed to optimize the performance of several color-difference formulas [26,27]. A color-difference formula can be modified by the addition of an exponent *b* (power function), as shown in Equation (4), where ΔE may be any of the color-difference formulas shown in Equations (1)–(3), and *b* is a constant that is optimized to improve the fit of specific visual data:

(4) $$\Delta E^{\prime }=\Delta E^{b}$$

It has been pointed out that the usefulness of power functions in color difference evaluation is related to the performance of the human visual system when visual color differences are in a wide range of magnitude [28], as in the current study (see Figure 1). In fact, it is clear that the introduction of an exponent *b* in a color-difference formula calls for modifications of the color coordinates of the color space supporting that formula. The uniformity of color spaces has long been analyzed [37,38]; CIE is currently working to make proposals (e.g., CIE TC 1-98 may soon suggest researching an approximately uniform *LMS*-based color space similar to CIELAB). 

For the eight color differences tested in the current study, Table 2 shows the values of exponent *b* that were proposed in previous papers [8,26,27] based on experimental visual results obtained using 2D samples with very different materials. In the case of the CAM16-(LCD/SCD/UCS) formulas, exponent *b* was only fitted to the CAM16-UCS formula [8]. Our current goal was to determine the optimal values of exponent *b* when these color-difference formulas were used to predict visual results for 3D samples in [24]. 

## 3. Results and Discussion

### 3.1. Performance of the Original Color-Difference Formulas 

Table 3 shows the *STRESS* values [25] for the eight color-difference formulas considered in the current study (CIELAB, CIEDE2000, CAM02-(LCD/SCD/UCS), and CAM16-(LCD/SCD/UCS)), for each of the eight phases (see Table 1, column 1) in the experiments using 3D objects [24] as well as for the combined visual results of such experiments, which included a total of 440 color pairs (see Table 1, phase 9). In Table 3, the lowest *STRESS* values for each phase are in bold to indicate the best color-difference formula.

For the combined dataset (Table 3, phase 9), the CAM16-LCD and CIELAB formulas had the best and worst performances, respectively. However, we noted that for phase 9 the *STRESS* values of the eight formulas were in a narrow range (27.3–31.9 *STRESS* units), which meant that all of the formulas performed similarly. In addition, this *STRESS* range was close to the inter-observer variability (30.3–31.2 *STRESS* units) reported in [24]. Table 4 shows the numbers of the phases in which the color-difference formula shown in the first row was significantly better statistically than the color-difference formula shown in the first column based on F-tests with two-tailed distributions and 95% confidence levels [25]. The empty cells in Table 4 signify no statistically significant improvements for the corresponding formulas in any phase. In agreement with the fact that the *STRESS* values for the eight color-difference formulas were quite similar (Table 3), Table 4 shows that very few formulas (10/56) were significantly better statistically than others in some phase/s. Specifically, for the combined dataset (phase 9), we can see that only CAM02-(LCD-SCD-UCS) and CAM16-(LCD-SCD-UCS) were significantly better statistically than CIELAB, and that CAM16-LCD was significantly better statistically than CIEDE2000. 

### 3.2. Optimizations of Color-Difference Formulas

Table 3 illustrates the accuracy of the predictions of the visually perceived color differences of 3D objects [24] made by eight color-difference formulas developed using 2D objects. In the current subsection, we will improve the performance of each one of these formulas. That is, we will minimize the *STRESS* values shown in Table 3 using the Excel Solver GRG non-linear method for the following three methods:

(1) Optimization of the value of the lightness parametric factor (*k_L_*) described in Section 2.3.1. For each phase and color-difference formula, Table 5 shows the optimal *k_L_* values that provided the minimum *STRESS* values, which are shown in Table 6. Table 5 indicates that most of the optimal *k_L_* values were slightly higher than 1.0. In any case, based on the comparison of the *STRESS* values in Table 3 and Table 6, the most important conclusion we reached was that the improvement achieved via the introduction of a lightness parametric factors was slight. Specifically, no color-difference formula achieved a statistically significant improvement in any phase with respect to the original formula via the introduction of the lightness parametric factor. For example, for phase 9 (the combined dataset), the average decrease in *STRESS* values for the eight color-difference formulas was only 2.6% (5.9% when considering the average of phases 1–8).
(2) Optimization of the value of exponent *b* (power function) described in Section 2.3.2. For each phase and color-difference formula, Table 7 shows the optimal *b* values that provided the minimum *STRESS* values, which are shown in Table 8. In Table 7, all of the optimal exponents have values lower than 1.0, which was in agreement with the previous literature [26,27] and were slightly higher than those shown in Table 2 for 2D objects. After comparing Table 3 and Table 8, we concluded that the introduction of exponents (power functions) produced a very important decrease in the *STRESS* values, which may be in part related to the use of color differences in a wide range of magnitude (see Figure 1). For example, for phase 9 (the combined dataset), the average decrease in the *STRESS* values for the eight color-difference formulas with the exponents shown in Table 7 was as high as 26.9% (29.7% when considering the average of phases 1–8). As a consequence, any of these eight color-difference formulas that was modified by the corresponding exponent was much better than the original formula.
(3) Simultaneous optimizations of the values of the lightness parametric factor (*k_L_*) and exponent (*b*). Table 9 shows the optimal *k_L_* and *b* values that provided the minimum *STRESS* values, which are shown in Table 10. In Table 9, we can note that most of the values of *k_L_* were slightly higher than those shown in Table 5, while the values for exponent *b* were almost the same as those shown in Table 7 (e.g., differences below 0.1 units for all phases and formulas), which were only slightly higher than those found for 2D objects (see Table 2). The *STRESS* values shown in Table 10 were slightly lower than those in Table 8, which meant that the simultaneous optimization of *k_L_* and *b* slightly improved the good results obtained by only optimizing exponent *b*. Specifically, after comparing Table 3 and Table 10, we concluded that based on the simultaneous optimization of *k_L_* and b, the average decrease in the *STRESS* values for the eight formulas in phase 9 (the combined dataset) was 29.6% (34.8% when considering the average of phases 1–8), and, once again, any of these modified color-difference formulas was much better than the original formula for phase 9 (and also for most cases in phases 1–8). We thus concluded that the *k_L_* and *b* values in Table 9 provided the best optimized color-difference formulas that can be proposed based on the visual results previously reported in [24].

For phase 9 (the combined dataset), the lowest *STRESS* values (i.e., best performances) among the tested color-difference formulas were found for CAM16-LCD in the case of the original formulas (Table 3) and formulas optimized by parametric factors *k_L_* (Table 6), CAM16-SCD for formulas optimized by exponents *b* (Table 8), and CAM16-UCS for formulas simultaneously optimized by *k_L_* and *b* (Table 10). These results were encouraging because they may suggest that CIECAM16 [9], the latest color appearance model proposed by the CIE, is a good basis for proposing future successful color-difference formulas for 3D objects.

Table 11 shows the results of the F-tests for the *STRESS* values [25] achieved by the best-optimized color-difference formulas (Table 10) with respect to the original formulas (Table 3). Although Table 11 shows that the number of cases with statistically significant differences was higher than in Table 4, the main conclusions we reached based on Table 4 and Table 11 were similar: (1) in most cases, the original (or modified) CIELAB color-difference formula was significantly worse statistically than any of the seven remaining original (or modified) color-difference formulas; and (2) in most cases, the differences among the original (or modified) CIEDE2000, CAM02-(LCD/SCD/UCS), and CAM16-(LCD/SCD/UCS) color-difference formulas were not statistically significant.

**Figure 2:**
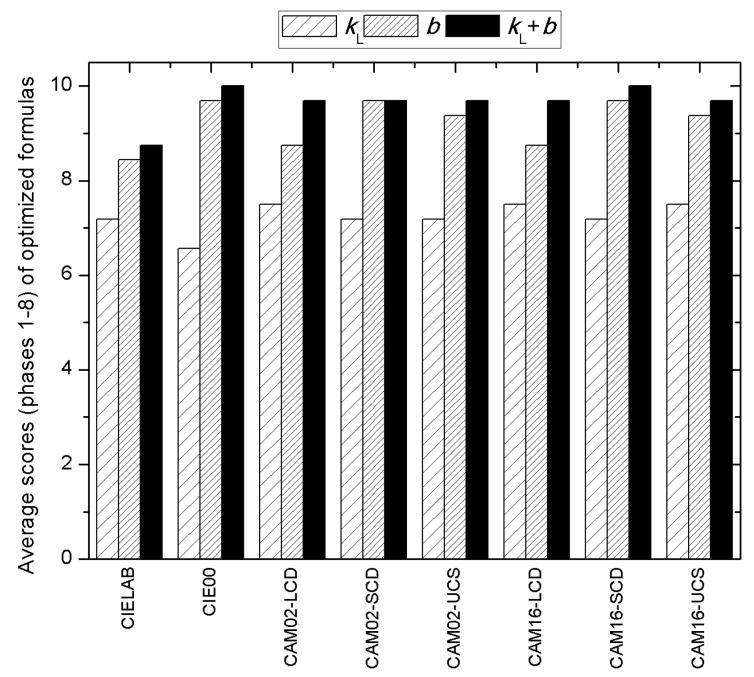
Average scores (phases 1–8) of eight color-difference formulas optimized by lightness parametric factors *k_L_* (Table 5), exponent *b* (Table 7), and *k_L_* + *b* (Table 9) with respect to the original formulas (Table 3). For instance, scores of 5 and 10 mean identical and significantly better performances statistically, respectively.

Figure 2 shows another way to compare the improvements achieved by each of the eight original color-difference formulas when modified using the three methods described above: the lightness parametric factor *k_L_* (Table 5); exponent *b* (Table 7); and the lightness parametric factor plus exponent *k_L_* + *b* (Table 9). Specifically, Figure 2 shows the average scores (phases 1–8) for each modified formula arbitrarily assigning 10, 7.5, 5, 2.5, and 0 points when a modified formula was statistically significantly better, better, identical, worse, or significantly worse, respectively, than the original formula. In Figure 2, it can be noted that maximum scores (10 points) were only obtained for the *k_L_* + *b* modified CIEDE2000 and CAM16-SCD formulas (see their corresponding *k_L_* and *b* parameters in Table 9). However, we must add that unfortunately, a general recommendation of either of these two color-difference formulas for 3D objects was not completely justified based on the current visual data [24]. As indicated above, a comparison among all of the *k_L_* + *b*-optimized formulas indicated no statistically significant differences between the CIEDE2000, CAM02-(LCD/SCD/UCS), and CAM16-(LCD/SCD/UCS) formulas in most cases (see Table 11). We could therefore only conclude that for the current experiments using 3D objects [24], the CIELAB color-difference formula (original or modified) was significantly worse statistically than the seven remaining (original or modified) formulas we tested. 

### 3.3. Parametric Effects

In the current section, we will discard the results from the CIELAB formula because in most cases it was found to be significantly worse statistically than the remaining seven color-difference formulas, as explained previously (see Section 3.1 and Section 3.2). We will therefore analyze the effect on the *STRESS* values of the CIEDE2000, CAM02-(LCD/SCD/UCS), and CAM16-(LCD/SCD/UCS) color-difference formulas according to changes in four parametric factors: color-difference magnitude, shape, gloss, and size of the 3D objects. The magnitude of color differences is an interesting factor because the CIEDE2000 formula was recommended for color-differences below 5.0 CIELAB units [4], while in the current experiments [24] there was a high percentage (45.1%) of color pairs with larger color differences (see Figure 1). Specific color-difference formulas for large color differences have been proposed in the literature [39]. We will also analyze the influence of shape (cones, spheres, and cylinders), gloss (3.6 vs. 96.6 GU on the average), and size (4 cm and 2 cm) on the *STRESS* values for the 3D samples studied. 

Table 12 shows the average *STRESS* changes for seven (original and *k_L_* + *b*-optimized) color-difference formulas for each of the four above-mentioned parametric factors. The footnote in Table 12 indicates the specific differences in the phases that we averaged to achieve the values shown in this table. All of the results in Table 12 came from differences in the *STRESS* values for pairs of phases (the order of such phases was relevant) in which only one parametric factor was changed at a time. For example, for the results in Table 12 regarding size, we computed the *STRESS* differences between phase 1 (Sp-4-m) and phase 3 (Sp-2-m) in that order, as well as the *STRESS* differences between phase 2 (Sp-4-g) and phase 4 (Sp-2-g) in that order; finally, we averaged both *STRESS* differences. That is to say, in this example of the effect of size, we considered differences between pairs of 3D samples with the same shape and gloss and with only a change from 4 cm to 2 cm (in that order). Therefore, the positive values for the parameter size shown in Table 12 for all color-difference formulas (except CAM16-LCD) mean higher *STRESS* values (i.e., lower performances) of the color-difference formulas for samples using 4 cm. 

In Table 12, we can note that in most cases the values for the *k_L_* + *b*-optimized formulas were lower than those corresponding to the original formulas. These lower *STRESS* differences may have been a consequence of the *STRESS* values for *k_L_* + *b*-optimized formulas (Table 10), which were considerably lower than those for the original formulas (Table 3), as discussed in Section 3.2. Based on the values in the last column of Table 12, we also concluded that the most important effects on the performance of original or *k_L_* + *b*-optimized formulas were the color-difference magnitude, shape, gloss, and size (in that order). We will make some comments on each one of these parametric factors in the paragraphs that follow.

Regarding the magnitude of color differences, Table 12 indicates that the seven original (or *k_L_* + *b*-optimized) formulas performed worse (i.e., had higher *STRESS* values) for color pairs with color differences below 5.0 CIELAB units. This result was not expected for CIEDE2000 because it was recommended for color pairs with color differences below 5.0 CIELAB units [4], although in fact pairs with color differences up to 18.2 CIELAB units were employed in the development of CIEDE2000.

The shape of 3D objects seemed to be another relevant parametric factor that produced variations in color in the samples, which were related to specific geometrical lighting and viewing conditions in the visual experiments in [24]. In the case here, cones and cylinders led to the highest and lowest *STRESS* values, respectively, with spheres adopting intermediate *STRESS* values. Specifically, for cone–cylinder pairs, we obtained on average 8.1 and 4.4 *STRESS* units of difference for the original and *k_L_* + *b*-optimized color-difference formulas, respectively; while for cone–sphere pairs, the average difference was 1.5 *STRESS* units for both types of formulas. A potential explanation for this result was that the visual contours for cylinders were rectangles (circles for spheres and nearly triangles for cones), which was the shape most similar to the 2D pairs of samples. The color gradients for the cones and spheres were also perhaps higher than for cylinders.

Gloss is an important perceptual factor that can strongly influence the visual perception of color differences [40,41]. The results for gloss given in Table 12 were the average of four pairs of phases (interested readers may find more detailed results in the *STRESS* values shown in Table 3 and Table 10); they suggested that the color-difference formulas tested performed slightly worse (i.e., had higher *STRESS* values) for the matte samples. However, interactions between the gloss and shape may have existed. 

Finally, based on the data in Table 12, the size of the samples seemed to be the least influential parametric factor in the performance of the tested color-difference formulas. Perhaps the change in size from 4 to 2 cm at a viewing distance of 40 cm was too small to produce relevant changes in the perception of color differences. In any case, there also seemed to be some kind of interaction between the size and gloss, as our results (not shown in Table 12) indicated that for the matte samples, the performance of most of the color-difference formulas was worse for the 4 cm size than for the 2 cm size, with the opposite being true for the gloss samples. 

To evaluate the statistical significance of the four above-mentioned parametric factors on the performance of the color-difference formulas tested, we carried out a statistical analysis that was different from the conventional F-tests [25]. The usual F-tests [25,30] require the same visual data be evaluated using two different color-difference formulas, while here we had pairs of phases rather than the same visual data. Using Student’s *t*-tests, we obtained no statistically significant differences for any of the four tested parametric factors using any of the (original or *k_L_* + *b*-optimized) color-difference formulas, which was in part expected because the values shown in Table 12 are small and the amount of data (pairs of phases) was very small in our study.

## 4. Conclusions and Future Work

Based on the visual data obtained for 3D-printed objects in a previous study [24], we used the *STRESS* index [25] to test the performances of eight color-difference formulas (CIELAB, CIEDE2000, CAM02-LCD, CAM02-SCD, CAM02-UCS, CAM16-LCD, CAM16-SCD, and CAM16-UCS) developed in experiments with 2D objects. Three optimizations of each one of these formulas were tested: the introduction of a lightness parametric factor (*k_L_*), which produced a small improvement over the original formulas; the introduction of an exponent (*b*), which produced a great improvement over those formulas; and the introduction of both *k_L_* and *b*, which led to the best-optimized formulas (i.e., the ones with the lowest *STRESS* values). 

In most cases, the original (or modified) CIELAB color-difference formula was significantly worse statistically than the remaining original (or modified) color-difference formulas. However, unfortunately, the differences among the original (or modified) CIEDE2000, CAM02-LCD, CAM02-SCD, CAM02-UCS, CAM16-LCD, CAM16-SCD, and CAM16-UCS formulas were not statistically significant, and thus it was not possible to recommend only one of these formulas for predictions regarding the color differences of 3D objects. Therefore, the use of any one of these seven formulas with the values of parameters *k_L_* and *b* shown in phase 9 of Table 9 is recommended. Obviously, further experimental results using 3D object colors are needed to improve the current recommendation.

Finally, we analyzed the influence of four parametric factors (color-difference magnitude, shape, gloss, and size of the 3D objects) on the performance (i.e., the *STRESS* values) of the CIEDE2000, CAM02-LCD, CAM02-SCD, CAM02-UCS, CAM16-LCD, CAM16-SCD, and CAM16-UCS original and *k_L_* + *b*-optimized formulas. None of these parametric effects had a statistically significant influence on the performances of these formulas, but the most influential ones were color-difference magnitude, shape, gloss, and size (in that order).

The results of the current investigation only apply to the few specific 3D objects considered in [24]. It was beyond the scope of this paper to generalize the current conclusions to 3D objects with any shape, gloss, size, etc. It must be also noted that the results reported here were based on values found using the *STRESS* index, which is currently recommended by the CIE [25] to compare the merit of color-difference formulas. Analyses of results achieved using other proposed indices [42] such as, for example, the gamma index [43], were outside of the scope of this paper. The effectiveness of the exponent (power factor) *b* also needs to be further analyzed because it introduces compression and homoscedasticity, which influence the results provided by the *STRESS* index. Finally, we also believe that the development of improved color-difference formulas for industrial applications requires new experimental visual datasets, as requested by CIE [3,17].

## Figures and Tables

**Table 1:** Main characteristics of the eight phases in the experiments reported in [24].

Phase	Samples	Material	Size (cm)	Number of Pairs	Range of $\Delta E_{ab,10}^{*}$	Average $\Delta E_{ab,10}^{*}$	Number of Observers	Total Visual Assessments
1	Sp-4-m	Matte	4	150	0.8–12.7	5.0	33	6450
2	Sp-4-g	Gloss	4	50	0.5–13.1	4.7	26	2500
3	Sp-2-m	Matte	2	40	0.5–16.9	5.5	45	5880
5	Co-4-m		4	40		5.2		
7	Cy-4-m		4	40		6.2		
4	Sp-2-g	Gloss	2	40	0.7–13.5	5.1	35	5880
6	Co-4-g		4	40		5.3		
8	Cy-4-g		4	40		5.2		
9	Combined	-	-	440	0.5–16.9	5.2	45	20,170

**Table 2:** Proposed values of exponent *b* for 2D samples in several color-difference formulas [8,26,27].

Exponent	CIELAB	CIEDE2000	CAM02-			CAM16-		
			LCD	SCD	UCS	LCD	SCD	UCS
*b*	0.55	0.70	0.85	0.75	0.75	/	/	0.63

**Table 3:** *STRESS* values [25] for eight color-difference formulas considering each of the nine phases (Table 1) in the visual experiments with 3D objects reported in [24]. The numbers in bold indicate the best color-difference formula (lowest *STRESS* value) for each phase.

Phase	Samples	CIELAB	CIEDE2000	CAM02-			CAM16-		
				LCD	SCD	UCS	LCD	SCD	UCS
1	Sp-4-m	31.6	33.4	28.4	30.3	29.0	**27.8**	30.2	28.7
2	Sp-4-g	25.8	**25.4**	25.5	25.5	25.7	25.5	25.9	26.0
3	Sp-2-m	31.9	**26.9**	27.2	28.7	27.3	27.6	28.5	27.4
4	Sp-2-g	34.2	29.1	26.6	25.7	**25.0**	27.7	27.2	26.3
5	Co-4-m	30.3	29.7	30.8	30.8	30.8	30.5	**29.7**	30.0
6	Co-4-g	**26.4**	27.6	27.6	28.4	27.5	28.6	28.5	28.1
7	Cy-4-m	29.3	26.3	**17.5**	24.5	20.3	18.4	23.2	20.2
8	Cy-4-g	36.9	19.5	24.0	20.4	20.0	22.9	19.2	**18.9**
9	Combined	31.9	30.0	27.4	29.0	27.7	**27.3**	28.6	27.5

**Table 4:** F-test results [25] from values in Table 3 showing the numbers of the phases (Table 1) with statistically significant improvements of the color-difference formula in the first row with respect to that in the first column.

	CIELAB	CIEDE2000	CAM02-LCD	CAM02-SCD	CAM02-UCS	CAM16-LCD	CAM16-SCD	CAM16-UCS
CIELAB	-	8	7, 8, 9	8, 9	7, 8, 9	7, 8, 9	8, 9	7, 8, 9
CIEDE2000		-	1, 7			1, 7, 9		
CAM02-LCD			-					
CAM02-SCD			7	-				
CAM02-UCS					-			
CAM16-LCD						-		
CAM16-SCD							-	
CAM16-UCS								-

1 = Sp-4-m; 7 = Cy-4-m; 8 = Cy-4-g; 9 = combined.

**Table 5:** Optimized values of the lightness parametric factor *k_L_* for eight color-difference formulas and nine phases (see Table 1) in the visual experiments in [24].

Phase	Samples	CIELAB	CIEDE2000	CAM02-			CAM16-		
				LCD	SCD	UCS	LCD	SCD	UCS
1	Sp-4-m	0.85	0.90	1.06	1.19	1.14	1.04	1.14	1.1
2	Sp-4-g	0.94	1.28	1.29	1.78	1.63	1.26	1.73	1.58
3	Sp-2-m	0.81	1.02	1.02	1.11	1.08	1.01	1.12	1.07
4	Sp-2-g	0.71	1.03	1.18	1.37	1.33	1.18	1.35	1.32
5	Co-4-m	0.98	1.36	1.67	1.75	1.75	1.67	1.79	1.77
6	Co-4-g	1.18	1.21	1.46	1.39	1.42	1.49	1.45	1.47
7	Cy-4-m	0.56	0.75	0.84	0.89	0.88	0.88	0.92	0.92
8	Cy-4-g	0.51	0.98	0.87	1.13	1.08	0.95	1.18	1.15
9	Combined	0.80	0.98	1.11	1.23	1.20	1.12	1.23	1.20

**Table 6:** *STRESS* values [25] achieved by eight color-difference formulas based on values of the lightness parametric factor *k_L_* shown in Table 5. Numbers in bold indicate the best color-difference formula for each phase.

Phase	Samples	CIELAB	CIEDE2000	CAM02-			CAM16-		
				LCD	SCD	UCS	LCD	SCD	UCS
1	Sp-4-m	31.2	33.2	26.7	30.3	28.7	**26.3**	30.0	28.5
2	Sp-4-g	25.7	24.3	**20.4**	23.2	21.3	21.0	24	22.2
3	Sp-2-m	31.5	26.9	**25.8**	28.4	27.2	26.3	28.3	27.3
4	Sp-2-g	33.1	29.1	23.8	25.6	**23.4**	25.2	27.1	24.9
5	Co-4-m	30.3	28.3	23.3	29.3	26.7	**23.0**	27.8	25.6
6	Co-4-g	26.1	26.9	**21.3**	28.2	25.5	22.0	28.1	25.6
7	Cy-4-m	24.4	24.6	**17.3**	22.1	19.9	18.1	21.4	20.1
8	Cy-4-g	33.9	19.5	23.9	20.3	20.0	22.4	19.2	**18.6**
9	Combined	31.4	30.0	25.3	29	27.2	**25.2**	28.6	26.9

**Table 7:** Optimized values of the exponent *b* (power function) for the eight color-difference formulas and nine phases (see Table 1) in the visual experiments in [24].

Phase	Samples	CIELAB	CIEDE2000	CAM02-			CAM16-		
				LCD	SCD	UCS	LCD	SCD	UCS
1	Sp-4-m	0.57	0.53	0.61	0.57	0.59	0.61	0.57	0.59
2	Sp-4-g	0.61	0.59	0.60	0.59	0.59	0.61	0.59	0.59
3	Sp-2-m	0.5	0.51	0.52	0.50	0.51	0.52	0.50	0.51
4	Sp-2-g	0.53	0.57	0.62	0.61	0.63	0.6	0.59	0.61
5	Co-4-m	0.57	0.53	0.61	0.57	0.59	0.61	0.57	0.59
6	Co-4-g	0.74	0.61	0.63	0.59	0.61	0.62	0.59	0.60
7	Cy-4-m	0.55	0.54	0.68	0.57	0.62	0.67	0.59	0.62
8	Cy-4-g	0.49	0.67	0.70	0.68	0.72	0.72	0.69	0.73
9	Combined	0.57	0.56	0.62	0.58	0.60	0.61	0.58	0.60

**Table 8:** *STRESS* values [25] achieved by the eight color-difference formulas based on the values of exponent *b* (power function) shown in Table 7. Numbers in bold indicate the best color-difference formula for each phase.

Phase	Samples	CIELAB	CIEDE2000	CAM02-			CAM16-		
				LCD	SCD	UCS	LCD	SCD	UCS
1	Sp-4-m	25.5	21.8	22.4	20.8	21.2	21.5	**20.1**	20.5
2	Sp-4-g	18.3	**16.1**	18.5	16.5	17.5	18.6	16.4	17.4
3	Sp-2-m	20.7	**15.9**	17.3	16.4	16.4	17.5	16.3	16.4
4	Sp-2-g	27.0	17.0	21.0	**16.5**	17.9	21.4	17.1	18.5
5	Co-4-m	25.9	**20**	23.2	21.0	21.8	23.2	20.5	21.5
6	Co-4-g	25	**20.1**	22.6	20.2	21.0	23.1	20.3	21.3
7	Cy-4-m	22	12.5	12.4	13.0	11.8	12.7	13.2	**11.5**
8	Cy-4-g	29.7	**12.7**	21.1	15.1	16.5	20.1	14.1	15.5
9	Combined	25.8	19.8	21.7	19.8	20.2	21.3	**19.3**	19.8

**Table 9:** Simultaneously optimized values of the lightness parametric factor *k_L_* and exponent *b* (power function) for the eight color-difference formulas and nine phases (Table 1) in the visual experiments in [24].

Phase	Exp.		CIELAB	CIEDE2000	CAM02-			CAM16-		
					LCD	SCD	UCS	LCD	SCD	UCS
1	Sp-4-m	*k_L_*	0.91	1.11	1.16	1.45	1.34	1.19	1.44	1.35
		*b*	0.58	0.53	0.63	0.56	0.59	0.63	0.56	0.58
2	Sp-4-g	*k_L_*	0.97	1.73	1.39	2.22	1.91	1.36	2.23	1.90
		*b*	0.61	0.59	0.68	0.62	0.65	0.67	0.61	0.64
3	Sp-2-m	*k_L_*	0.89	1.14	1.16	1.32	1.26	1.15	1.32	1.25
		*b*	0.50	0.51	0.55	0.50	0.52	0.54	0.49	0.51
4	Sp-2-g	*k_L_*	0.73	1.27	1.23	1.65	1.51	1.26	1.70	1.55
		*b*	0.54	0.57	0.66	0.61	0.65	0.64	0.59	0.62
5	Co-4-m	*k_L_*	0.89	1.76	1.65	2.04	1.90	1.67	2.05	1.92
		*b*	0.60	0.54	0.70	0.57	0.61	0.70	0.58	0.62
6	Co-4-g	*k_L_*	1.17	1.69	1.66	1.96	1.84	1.70	2.05	1.92
		*b*	0.75	0.6	0.72	0.57	0.62	0.70	0.58	0.62
7	Cy-4-m	*k_L_*	0.54	0.93	0.84	1.01	0.95	0.90	1.03	1.01
		*b*	0.62	0.54	0.68	0.59	0.63	0.67	0.61	0.62
8	Cy-4-g	*k_L_*	0.45	1.30	0.87	1.33	1.19	0.95	1.39	1.26
		*b*	0.54	0.65	0.71	0.68	0.71	0.72	0.69	0.72
9	Combined	*k_L_*	0.81	1.23	1.19	1.51	1.4	1.23	1.54	1.44
		*b*	0.58	0.55	0.65	0.57	0.60	0.64	0.57	0.60

**Table 10:** *STRESS* values [25] achieved by the eight color-difference formulas based on the values of *k_L_* and *b* shown in Table 9. The numbers in bold indicate the best color-difference formula for each phase.

Phase	Samples	CIELAB	CIEDE2000	CAM02-			CAM16-		
				LCD	SCD	UCS	LCD	SCD	UCS
1	Sp-4-m	25.5	21.7	21.0	20.6	20.4	20.0	20.0	**19.7**
2	Sp-4-g	18.3	13.3	14.8	13.2	**13.1**	15.3	13.2	13.4
3	Sp-2-m	20.7	**15.7**	16.0	16.3	15.9	16.4	16.2	16.0
4	Sp-2-g	26.6	16.4	19.3	**15.6**	16.0	19.8	16.1	16.6
5	Co-4-m	25.8	**17.9**	19.6	19.6	19.2	19.5	18.9	18.7
6	Co-4-g	24.8	18.1	**17.6**	18.7	18	18.0	18.5	18.0
7	Cy-4-m	19.6	12.4	12.3	12.5	11.8	12.4	12.8	**11.5**
8	Cy-4-g	28.1	**12.2**	21.1	15.1	16.3	19.8	13.9	15.0
9	Combined	25.6	19.5	20.2	19.5	19.3	19.7	19.0	**18.8**

**Table 11:** Results from F-tests [25] showing the phases with statistically significant improvements of the modified color-difference formulas obtained using the parameters shown in Table 9 with respect to the original formulas (Table 3). Only for the phases indicated were the formulas in the first row significantly better statistically than the formulas in the first column.

Modified Original	CIELAB	CIEDE2000	CAM02-LCD	CAM02-SCD	CAM02-UCS	CAM16-LCD	CAM16-SCD	CAM16-UCS
CIELAB	-	1, 2, 4, 5, 7, 8, 9	1, 4, 6, 7, 9	1, 2, 4, 7, 8, 9	1, 2, 4, 6, 7, 8, 9	1, 6, 7, 8, 9	1, 2, 4, 7, 8, 9	1, 2, 4, 5, 6, 7, 8, 9
CIEDE2000		-						
CAM02-LCD		8	-	8			8	8
CAM02-SCD				-				
CAM02-UCS					-			
CAM16-LCD		8				-	8	
CAM16-SCD							-	
CAM16-UCS								-

1 = Sp-4-m; 2 = Sp-4-g; 4 = Sp-2-g; 5 = Co-4-m; 6 = Co-4-g; 7 = Cy-4-m; 8 = Cy-4-g; 9 = combined.

**Table 12:** Differences in *STRESS* values from original (Table 3) and modified (*k_L_* + *b*) color-difference formulas (Table 10) while considering the following four parametric factors: color-difference magnitude, shape, gloss, and size of 3D samples (Table 1).

Color-Difference Formulas	Parametric Factors	CIEDE2000	CAM02-			CAM16-			Average
			LCD	SCD	UCS	LCD	SCD	UCS	
Original formulas (Table 3)	Magnitude ^1^	8.3	9.3	8.7	9.4	9.7	9.2	9.8	9.2
	Shape ^2^	3.8	5.6	4.8	6.0	5.9	5.3	6.3	5.4
	Gloss ^3^	3.7	0.0	3.6	2.3	−0.1	2.7	1.8	2.0
	Size ^4^	1.4	0.0	0.7	1.2	−1.0	0.2	0.5	0.4
Optimized (*k_L_* + *b*) formulas (Table 10)	Magnitude ^1^	5.3	6.9	5.5	6.0	7.4	6.1	6.5	6.2
	Shape ^2^	3.8	1.3	3.6	3.0	1.8	3.6	3.4	2.9
	Gloss ^3^	1.9	−1.0	1.6	1.0	−1.2	1.6	0.7	0.7
	Size ^4^	1.5	0.3	1.0	0.8	−0.4	0.4	0.2	0.5

^1^ Pairs with color differences below 5 CIELAB units−pairs with color differences above 5 CIELAB units. ^2^ Average of phases 5−1 and 6−2 (cone−sphere), 1−7 and 2−8 (sphere−cylinder), and 5−7 and 6−8 (cone−cylinder). ^3^ Average of phases 1−2, 3−4, 5−6, and 7−8; i.e., matte–gloss samples. ^4^ Average of phases 1−3 and 2−4; i.e., 4 cm–2 cm.

## Data Availability

The data underlying the results presented in this paper are not publicly available at this time but may be obtained from the authors upon reasonable request.
